# BRAIN GLUTATHIONE, MITOCHONDRIA, GLUCOSE METABOLISM, AND COGNITION IN AGED MICE: IMPACT OF GLYNAC SUPPLEMENTATION

**DOI:** 10.1093/geroni/igae098.0808

**Published:** 2024-12-31

**Authors:** Premranjan Kumar, Ob Osahon, Rajagopal Sekhar

**Affiliations:** Baylor College of Medicine, Houston, Texas, United States; Baylor College of Medicine, Houston, Texas, United States; Baylor College of Medicine, Houston, Texas, United States

## Abstract

Alzheimer’s disease (AD) remains unsolved, and is associated with brain defects including mitochondrial dysfunction, impaired glucose uptake, inflammation, and glutathione deficiency. These defects also occur in ‘age-related cognitive decline’ (ACD). Mouse models have been developed to study AD, however because mice do not develop AD naturally, these models are artificial as defects are induced in mice. To understand the mechanistic basis of cognitive decline in aging, it is important to investigate naturally-occurring defects directly in the brain, and this is only possible in animal models because performing brain biopsies in humans is not practical. To overcome these challenges, we studied mitochondrial function, glucose uptake, inflammation, redox status, genomic damage and neurotropic factors directly in the brains of old-mice (OM, 98-weeks) and young-mice (YM, 28-weeks), and also measured their cognitive performance (via maze-running). Compared to YM, the cognitively impaired OM had the severe defects in the brain: (a) lower glutathione concentrations; (b) elevated oxidative stress; (c) abnormal immunoblot expressions of: lower GLUT1 and GLUT3 (transporters regulating glucose uptake); lower PGC1a; electron-chain complexes and ATP5A (mitochondrial dysfunction); lower LC3A/B and PINK1 (autophagy/mitophagy); higher TSPO (inflammation), higher pH2AX (genomic damage), and lower BDNF, GDNF and NGF (neurotropic factors); (d) impaired mitochondrial respiration (respirometry); (e) impaired cognition (maze-time). After 8-weeks of supplementing GlyNAC (combination of glutathione precursors glycine and N-acetylcysteine), all defects improved/corrected in the brains of OM, and cognitive performance improved significantly. Study findings suggest that correcting glutathione deficiency could be important for improving brain health and cognition in ACD and in AD.

